# Perceived Social Support and Well-Being: Mediation and Buffering of the Stress–Depression Link in Rural Older Adults

**DOI:** 10.3390/healthcare14030336

**Published:** 2026-01-29

**Authors:** Paul Alan Arkin Alvarado-García, Taniht Lisseth Cubas Romero, Lis Paola Reyes Sánchez, Valeria Alexxandra Sandoval Bocanegra, Marilú Roxana Soto-Vásquez

**Affiliations:** 1 Escuela de Psicología, Universidad Autónoma del Perú, Lima 15842, Peru; taniht.cubas@autonoma.pe; 2 Puesto de Salud Pinaya, Puno 21770, Peru; lisreyessa@gmail.com; 3 Puesto de Salud Soila Victoria Núñez, Cajamarca 06121, Peru; vsandovalbocanegra@gmail.com; 4 Facultad de Farmacia y Bioquímica, Universidad Nacional de Trujillo, Trujillo 13011, Peru; msoto@unitru.edu.pe

**Keywords:** perceived social support, psychological stress, depressive symptoms, psychological well-being, older adults, rural population, Peru, Latin America

## Abstract

**Background/Objectives:** Rural older adults are exposed to multiple chronic stressors that may heighten depressive symptoms, and these effects can be intensified by social disconnection, particularly in resource-constrained settings. This study examined whether global and dimension-specific perceived social support—an indicator of perceived social connection—mediates and/or buffers the association between perceived stress and depressive symptoms in rural older adults from northern Peru. **Methods:** A cross-sectional survey was conducted with 166 community-dwelling adults aged ≥60 years in a rural coastal district. Perceived stress (PSS-4), depressive symptoms (GDS-15), and perceived social support (MOS-SSS) were assessed. Regression-based mediation and moderation models with bootstrapped confidence intervals were estimated, adjusting for age, sex, marital status, education, income category, and chronic medical conditions. **Results:** Higher perceived stress was associated with greater depressive symptoms. Greater overall social support was associated with lower perceived stress and fewer depressive symptoms. Indirect effects supported a stress-process pathway for overall support, particularly socioemotional dimensions (positive social interaction and affectionate support). No buffering effect was observed for overall support; however, tangible (instrumental) support attenuated the association between stress and depressive symptoms. **Conclusions:** Mediation analyses supported an indirect pathway linking perceived stress to depressive symptoms via socioemotional support, whereas tangible (instrumental) support moderated the stress–depression association. Interventions that strengthen social connectedness and practical assistance may help protect mental health in rural older adults.

## 1. Introduction

Population aging is accelerating worldwide, especially in low- and middle-income countries, and maintaining psychological well-being in later life has become a significant public health priority [1]. According to the World Health Organization (WHO), the global population aged 60 years and older will increase from 1.0 billion in 2020 to 1.4 billion by 2030 and is projected to double to 2.1 billion by 2050, with two-thirds living in low- and middle-income countries [2]. Depressive disorders remain among the leading contributors to non-fatal health loss globally [3], and Late-life depression is associated with impaired quality of life, functional decline, and increased use of health services [4]. The WHO estimates that around 14% of adults aged 70 years and older live with a mental disorder, with depression and anxiety being the most prevalent [5]. These burdens are often magnified among older adults with limited financial resources, chronic medical conditions, and restricted access to services [6,7], including many who live in rural and disadvantaged areas [8].

Moreover, psychological stress is a significant risk factor for depressive symptoms in later life. Evidence from longitudinal studies shows that higher perceived stress predicts more severe and persistent depressive symptoms in older adults [9,10], and similar associations have been reported in cohort research [11]. Stressful experiences and chronic strain, such as multimorbidity, caregiving, or economic hardship, can tax older adults’ emotional and physiological resources, heightening vulnerability to depression and other common mental disorders [12,13]. However, not all highly stressed older adults develop clinically significant depressive symptoms, suggesting the presence of protective resources that shape how stress translates into mental health outcomes.

Social connections and perceived social support are widely recognized as core protective resources in later life. Perceived social support, defined as the belief that one is cared for, valued, and can count on others when needed, has been consistently linked to lower levels of depressive symptoms and better psychological health among older adults [14]. Evidence across multiple settings consistently shows that older adults reporting higher social support or stronger family connections are less prone to late-life depression and severe depressive symptoms [15,16]. Perceived support is also closely intertwined with loneliness and social disconnection, which have emerged as critical determinants of late-life mental health and well-being [17,18].

Importantly, social support is not a unitary construct. Multidimensional instruments distinguish emotional support (expression of empathy and understanding), positive social interaction or companionship, affectionate support, and instrumental or tangible assistance [19,20]. These dimensions may influence the stress–depression pathway through partially distinct mechanisms. Socio-emotional ties, such as companionship, affection, and a sense of closeness, can buffer stress appraisals and mitigate feelings of loneliness, hopelessness, or loss of meaning [21,22]. In contrast, tangible support, including assistance with transportation, finances, or daily tasks, may reduce practical demands and lessen the impact of stressors related to resource scarcity and functional limitations, while also enabling more effective problem-focused coping through instrumental aid and active coping assistance [23]. Consistent with this differentiation, evidence indicates that the protective value of support types is not uniform in later life [24], and a collaborative individual-participant data meta-analysis of 11 aging cohorts demonstrated that only emotional support—rather than instrumental support—was associated with lower depressive symptoms both cross-sectionally and longitudinally [25].

Two major theoretical perspectives specify how social support can protect mental health under stress. The classic stress-buffering hypothesis conceptualizes social support as a moderator: support attenuates, or buffers, the association between stress and adverse outcomes so that stress has weaker emotional consequences at higher levels of support [26,27]. In contrast, stress process models emphasize social support as a mediator: chronic stressors erode social relationships and perceived support over time, and this deterioration of social resources contributes to the development or worsening of depressive symptoms [28]. Both mechanisms are plausible and may operate simultaneously. Yet, empirical findings have been mixed regarding whether support primarily mediates, moderates, or does both, and whether specific dimensions of social support are differentially implicated in these processes [14,20,29].

These questions are especially salient in rural and low-income communities in Latin America, where older adults often face a combination of socioeconomic adversity, multimorbidity, and limited access to formal healthcare and social protection [30,31]. At the same time, family networks, neighborhood ties, and community organizations can provide crucial sources of both socio-emotional connection and instrumental assistance. Evidence from Latin American and Caribbean settings indicates that family support and social capital are closely linked to late-life depression [15,32]. In addition, studies from rural Andean and Amazonian contexts show that older adults in rural areas often experience high levels of common mental disorders in settings where formal services are limited [32,33]. Nevertheless, empirical studies that simultaneously model both mediating and buffering roles of multidimensional social support in relation to stress and depressive symptoms among rural older adults in the region remain scarce.

In this study, we focus on depressive symptoms as a key indicator of psychological well-being in older adults and investigate how perceived social support relates to the stress–depression association in a rural district of northern Peru. Specifically, we addressed three questions: (1) whether higher perceived stress is associated with greater depressive symptoms; (2) whether global perceived social support and its core dimensions (emotional, instrumental, positive social interaction, affectionate) help explain this association as mediators; and (3) whether these exact support dimensions attenuate, or buffer, the impact of stress on depressive symptoms as moderators. Based on stress process and stress-buffering models, we hypothesized that higher stress would be linked to more depressive symptoms, that lower perceived support, particularly in socio-emotional dimensions, would partly account for this link, and that instrumental (tangible) support would weaken the association between stress and depression. While reciprocal influences are also theoretically plausible (for example, depressive symptoms may erode perceived support and heighten stress), in this cross-sectional study we adopt a conceptual model in which stress is treated as the primary upstream predictor of depressive symptoms and perceived social support is examined as a potential social-connection mechanism in this pathway, acknowledging that alternative directions of effect cannot be ruled out.

## 2. Materials and Methods

### 2.1. Study Design and Participants

This cross-sectional, correlational study was conducted in a rural coastal district of northern Peru. Participants were 166 community-dwelling older adults, recruited by convenience sampling from a local primary health center. Data were collected in person using structured interviews. Eligible individuals were: (a) aged ≥60 years, (b) permanent residents of the selected rural area, and (c) able and willing to provide informed consent. Older adults with evident cognitive impairment, a known psychotic or severe psychiatric disorder, or current use of antidepressant or anxiolytic medication were excluded.

### 2.2. Sample Size Calculation

The required sample size for the planned mediation and moderation analyses was estimated using G*Power 3.1, assuming a linear multiple regression model. We targeted a medium effect size (f^2^ = 0.15), a two-sided α = 0.05, and high statistical power (1 − β = 0.99), with one focal predictor (e.g., the mediator or moderator) and up to eight predictors in the model (including stress, social support dimensions, interaction terms, and sociodemographic covariates). Under these assumptions, the minimum required sample size was 125 participants. The final sample of 166 older adults, therefore, exceeded this requirement and provided adequate power to detect effects of interest.

### 2.3. Measurements and Instruments

#### 2.3.1. Perceived Stress

Perceived stress was assessed using the 4-item Perceived Stress Scale (PSS-4), which evaluates the degree to which situations in one’s life are appraised as stressful over the past month. Items are rated on a 5-point Likert scale from 0 (“never”) to 4 (“very often”), and the scores are summed to yield a total score ranging from 0 to 16, with higher scores indicating greater perceived stress [34]. Spanish versions of the full Perceived Stress Scale have shown adequate psychometric properties in adult populations, including Peruvian samples [35,36,37]. In the present sample of rural older adults, the PSS-4 showed acceptable internal consistency (Cronbach’s α = 0.73), with corrected item–total correlations ranging from 0.22 to 0.90.

#### 2.3.2. Depressive Symptoms

Depressive symptoms were measured with the 15-item Geriatric Depression Scale (GDS-15), a widely used screening instrument for depression in older adults. Items are answered in a yes/no format and summed, with higher scores reflecting more severe depressive symptoms [38]. Spanish versions of the GDS-15 have demonstrated good reliability and validity among older adults in Latin America [39,40]. In this sample, corrected item–total correlations for the GDS-15 items were all greater than 0.24, and internal consistency was acceptable (Cronbach’s α = 0.71).

#### 2.3.3. Perceived Social Support

Perceived social support was evaluated using the Medical Outcomes Study Social Support Survey (MOS-SSS), which yields a global support score and four subscale scores: emotional/informational support, tangible (instrumental) support, positive social interaction, and affectionate support. The instrument consists of 19 items rated on a 5-point scale from 1 (“never”) to 5 (“always”), indicating how often different types of support are available. Higher scores indicate greater perceived support. The MOS-SSS has been validated in Spanish-speaking populations, with good psychometric performance [41,42]. In the present sample, internal consistency was excellent for the total score (Cronbach’s α = 0.96) and good-to-excellent for the subscales (emotional/informational α = 0.94; tangible α = 0.81; affectionate α = 0.93; positive social interaction α = 0.92). Because very high α values may reflect item redundancy [43,44], item-level diagnostics were examined. Specifically, we report corrected item–total correlations (item–rest correlations) and Cronbach’s α if an item is deleted for each MOS-SSS item (Table S1). Corrected item–total correlations were generally strong for emotional/informational, affectionate, and positive social interaction items (0.742–0.835) and lower for tangible-support items (0.088–0.430). One tangible-support item showed a very low item–rest correlation (0.088), but it was retained to preserve the content validity of the tangible-support domain and to maintain comparability with the standard MOS-SSS scoring, consistent with the instrument’s multidimensional design. Deleting this item produced only a small change in the total-scale α (α if item deleted = 0.953), and the tangible subscale showed acceptable internal consistency (α = 0.81).

#### 2.3.4. Sociodemographic and Health Variables

Participants reported age (years), sex (men/women), marital status, education level, income category (low vs. middle), and the presence of at least one chronic medical condition (yes/no; e.g., hypertension, diabetes). For regression-based models, age was entered as a continuous covariate, whereas sex, income category, and chronic medical condition were entered as dichotomous covariates. Marital status and education level were treated as categorical covariates using indicator coding; category definitions are provided in Table 1.

### 2.4. Procedure

Authorization to conduct the study was obtained from the director of the local primary health center. Trained interviewers approached potentially eligible older adults during routine visits or community outreach activities and screened for inclusion and exclusion criteria. After explaining the study objectives and procedures, written informed consent was obtained from all participants or their legal representatives when necessary. Structured face-to-face interviews were conducted at participants’ homes or community centers using standardized protocols to ensure consistent and respectful administration of the questionnaires. Completed forms were anonymized and entered into a secure database, with quality checks applied. All 166 eligible participants who provided consent completed the interview and were included in the analytic sample. The study adhered to the ethical principles of the Declaration of Helsinki, and the protocol was approved by the Research Ethics Committee of the School of Medicine, Universidad César Vallejo, Trujillo, Peru (Approval number: 129-CEI-EPM-UCV-2023; 26 June 2023).

### 2.5. Statistical Analysis

Descriptive statistics were used to summarize sociodemographic and clinical characteristics. Missing data were examined prior to analysis; because item nonresponse was low due to interviewer-administered questionnaires, analyses were conducted using complete cases (listwise deletion) without imputation. Given the non-normal distribution of several variables, Spearman’s rank-order correlations were computed to examine bivariate associations among perceived stress, depressive symptoms, and social support scores.

To test the mediating role of perceived social support in the association between stress and depressive symptoms, we first estimated a simple mediation model with the global MOS-SSS score as mediator (corresponding to Model 4 in PROCESS). We then specified a parallel multiple mediation model including the four MOS-SSS subscales (emotional/informational, tangible, positive social interaction, affectionate support) as simultaneous mediators. Given the high intercorrelations among MOS-SSS subscales, we evaluated multicollinearity in a linear regression model that included perceived stress and the four MOS-SSS dimensions as predictors of depressive symptoms. Variance inflation factors ranged from 1.157 to 6.380 (tolerance = 0.157–0.864), indicating moderate multicollinearity among some dimensions. In all mediation models, perceived stress was entered as the independent variable and depressive symptoms as the dependent variable. To test the buffering (moderating) role of each support dimension, we estimated separate moderation models (PROCESS Model 1), in which the interaction between perceived stress and each social support dimension was used to predict depressive symptoms.

All models were adjusted for age, sex, educational level, household income, and presence of at least one chronic medical condition. Continuous predictors were mean-centered before computing interaction terms. Indirect effects and their 95% confidence intervals were estimated using non-parametric bootstrapping with 10,000 resamples. Two-sided *p*-values < 0.05 were considered statistically significant. Data were analyzed using SPSS version 31.0 (IBM Corp., Armonk, NY, USA) and the Hayes’ PROCESS macro for SPSS [45].

## 3. Results

To enhance clarity and maintain alignment with the study objectives, Section 3 (Results) is presented in a thematic sequence. Section 3.1 describes participant characteristics, followed by descriptive statistics and correlations in Section 3.2. Subsequently, models examining the role of perceived social support in the stress–depressive symptom association are reported, first using overall support (Section 3.3) and then analyzing specific support dimensions (Section 3.4).

### 3.1. Participant Characteristics

Table 1 summarizes the sociodemographic profile of the 166 rural older adults included in the study. Participants had a mean age of 68.5 ± 6.9 years. Men predominated (61.4%), and most respondents were married (44.0%) or single (26.5%). Educational attainment was low overall: nearly half (49.2%) had only primary schooling—either incomplete (20.5%) or completed (18.7%)—and 6.0% were illiterate; just 9.6% reported any higher education. Regarding household resources, 58.4% were classified as low-income and 41.6% as middle-income.

**Table 1:** Sociodemographic Characteristics of Participants.

Variable	Category	n (%) or Mean ± SD
Age (years)		68.49 ± 6.89
Sex	Men	102 (61.4%)
	Women	64 (38.6%)
Marital status	Single	44 (26.5%)
	Married	73 (44.0%)
	Cohabiting	10 (6.0%)
	Divorced	9 (5.4%)
	Widowed	30 (18.1%)
Education level	Incomplete primary education	34 (20.5%)
	Completed primary education	31 (18.7%)
	Incomplete secondary education	26 (15.7%)
	Completed secondary education	49 (29.5%)
	Incomplete higher education	3 (1.8%)
	Completed higher education	13 (7.8%)
	Illiterate	10 (6.0%)
Income category	Middle income	69 (41.6%)
	Low income	97 (58.4%)
Chronic medical condition	Yes	134 (80.7%)
	No	32 (19.3%)

### 3.2. Descriptive Characteristics and Intercorrelations of Study Variables

Table 2 presents the nonparametric correlation analysis, revealing significant associations among stress, depressive symptoms, and perceived social support in 166 participants. As expected, stress was strongly and positively correlated with depression (ρ = 0.644, *p* < 0.05), indicating that more depressed symptomatology is associated with higher degrees of perceived stress. Stress was also inversely correlated with overall perceived social support (ρ = −0.511, *p* < 0.05), as well as with emotional support (ρ= −0.490), positive social interaction (ρ = −0.571), and affective support (ρ = −0.571), suggesting that stress is linked to diminished perceptions of emotionally meaningful and relational support. Notably, instrumental support did not correlate significantly with stress (ρ = 0.090), highlighting a potential distinction between emotional versus practical support dimensions under stress. Depression also showed strong negative correlations with overall social support (ρ = −0.706) and with socioemotional dimensions, particularly emotional support (ρ = −0.713), positive social interaction (ρ = −0.749), and affective support (ρ = −0.724), reinforcing the centrality of emotionally resonant forms of support in mitigating depressive outcomes. In contrast, tangible (instrumental) support was not significantly correlated with depression (ρ = 0.066). Intercorrelations among the social support subscales were also high (e.g., ρ = 0.853 between positive social interaction and affective support), indicating strong internal coherence of the construct. These results highlight, in the framework of psychological stress, the diverse roles that support forms play; emotional and interpersonal aspects of social support are essential protective elements.

### 3.3. Examination of Overall Social Support as a Mediator and Moderator Between Stress and Depression

In a simple mediation model adjusting for age, sex, marital status, educational level, economic level, and chronic medical condition (Table 3), higher perceived stress was associated with lower global perceived social support (a path: B = −3.62, SE = 0.55, t = −6.55, *p* < 0.001). In turn, higher social support was associated with fewer depressive symptoms when controlling for stress and covariates (b path: B = −0.12, SE = 0.01, t = −9.27, *p* < 0.001). Perceived stress showed a strong positive total effect on depressive symptoms (c path: B = 1.03, SE = 0.11, t = 9.30, *p* < 0.001), and this effect was attenuated yet remained significant after accounting for social support (c′ path: B = 0.60, SE = 0.10, t = 5.94, *p* < 0.001). The bootstrapped indirect effect of stress on depressive symptoms through global social support was statistically significant (ab = 0.43, BootSE = 0.08, 95% BootCI [0.28, 0.60]), supporting a significant indirect effect through global social support; the direct effect remained significant after accounting for the mediator (c′ path). The model explained 23% of the variance in social support and 60% of the variance in depressive symptoms.

In addition, Table 4 shows the results of the moderation model testing whether global perceived social support buffers the association between perceived stress and depressive symptoms. Higher perceived stress was associated with higher levels of depressive symptoms (B = 0.62, SE = 0.11, t = 5.80, *p* < 0.001, 95% CI [0.41, 0.83]). In contrast, greater global social support was associated with fewer depressive symptoms (B = −0.12, SE = 0.01, t = −9.23, *p* < 0.001, 95% CI [−0.15, −0.09]), controlling for sociodemographic factors and chronic medical conditions. However, the Stress × Social support interaction term was not significant (B = 0.00, SE = 0.01, t = 0.58, *p* = 0.562, 95% CI [−0.01, 0.02]), indicating no evidence that global perceived social support moderated (i.e., buffered) the relationship between stress and depressive symptoms. The overall model accounted for 60% of the variance in depressive symptoms (R^2^ = 0.60).

### 3.4. Mediation and Moderation Analyses by Social Support Dimensions

Table 5 presents the results of the parallel multiple mediation model examining the specific social support dimensions through which perceived stress is related to depressive symptoms. The total indirect effect of stress on depression through the four social support dimensions combined was significant (B = 0.67, BootSE = 0.10, 95% BootCI [0.46, 0.88]), with a completely standardized indirect effect of 0.38 (BootCI [0.28, 0.48]). Among the individual dimensions, positive social interaction (B = 0.29, BootSE = 0.13, 95% BootCI [0.06, 0.57]; completely standardized indirect effect = 0.17, BootCI [0.04, 0.32]) and affectionate support (B = 0.22, BootSE = 0.10, 95% BootCI [0.03, 0.44]; completely standardized indirect effect = 0.13, BootCI [0.02, 0.25]) showed significant specific indirect effects, indicating that lower levels of these forms of support partly transmit the association between higher stress and greater depressive symptoms. In contrast, the indirect effects through emotional/informational support and tangible (instrumental) support were small and not statistically significant, as their bootstrap confidence intervals included zero.

**Figure 1:**
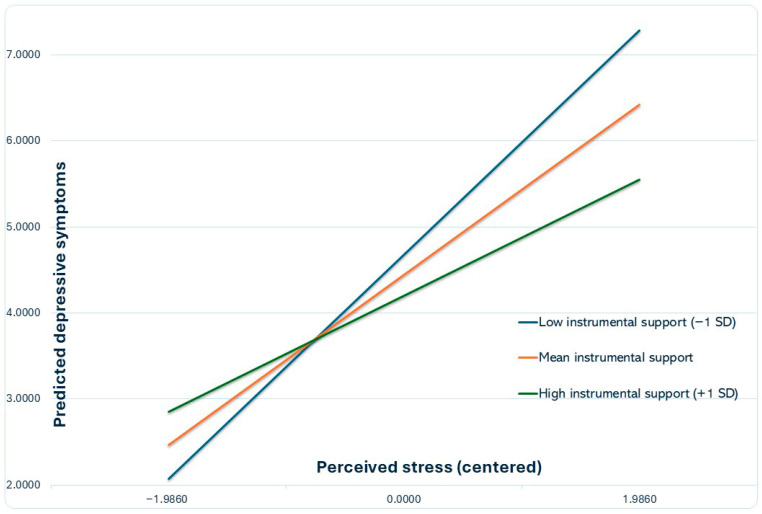
Stress-buffering effect of instrumental (tangible) support on depressive symptoms. Predicted depressive symptoms across levels of perceived stress at low (−1 SD), mean, and high (+1 SD) levels of instrumental support, adjusted for age, sex, educational level, household income, and chronic medical conditions. Stress and instrumental support were mean-centered prior to computing the interaction.

As shown in Table 6, tangible (instrumental) support significantly moderated the association between perceived stress and depressive symptoms (B = −0.0827, SE = 0.0370, t = −2.2356, *p* = 0.0268, 95% CI [−0.1558, −0.0096]), accounting for an incremental 1.9% of explained variance in depressive symptoms (ΔR^2^ = 0.0190, F(1156) = 4.9980, *p* = 0.0268). Simple slope analyses indicated that perceived stress was positively associated with depressive symptoms at low tangible support (b = 1.3115, SE = 0.1648, *p* < 0.001, 95% CI [0.9860, 1.6371]), at mean tangible support (b = 0.9958, SE = 0.1114, *p* < 0.001, 95% CI [0.7757, 1.2159]), and at high tangible support (b = 0.6801, SE = 0.1938, *p* = 0.0006, 95% CI [0.2972, 1.0629]), consistent with a stress-buffering pattern. Johnson–Neyman analysis indicated that the conditional effect of stress on depressive symptoms became non-significant when tangible support exceeded 5.8472 (mean-centered units), a level observed in approximately 9.0% of the sample. Figure 1 illustrates the interaction.

## 4. Discussion

The present study was conducted in a sugar-cane-producing district on Peru’s northern coast, where older adults live in scattered rural settlements that can constrain day-to-day social contact and increase vulnerability to social isolation and loneliness, despite being in a nominally “developed” coastal corridor, experience marked economic deprivation, limited health-service coverage, and rising community violence. Indeed, 25.9% of Peruvians remained below the poverty line in 2021, with rural coastal pockets lagging behind urban areas [46]. These communities document job insecurity, hazardous labor, and elevated mental-health burden [47], while escalating crime has been linked to persistent fear and psychological distress [48].

### 4.1. Stress, Depression, and Social Support in Rural Older Adults

In this context, we identified that perceived stress was positively associated with depressive symptoms, which is in agreement with evidence from large community-based cohorts in Asia and North America, where the same relationship was observed [9,49]. In fact, higher perceived stress is robustly associated with more severe depressive symptoms in older adults [32,50]; especially in rural settings, with economically disadvantaged, older adults often face multiple chronic stressors such as poverty, limited access to health services, and high multimorbidity, which have been repeatedly linked to elevated depressive symptoms and poorer well-being [51]. Our sample, drawn from a rural primary-care setting and characterized by low educational attainment and frequent chronic conditions, fits this profile and extends previous findings on stress and depression to an understudied Latin-American rural context.

In addition, stress was inversely related to global perceived social support and its socio-emotional dimensions, in line with extensive evidence that higher stress and lower support are associated with deteriorating mental health in later life [52]. We also found strong negative correlations between depressive symptoms and global perceived social support, particularly emotional, interactional, and affectionate support. This aligns with recent evidence that older adults with higher perceived support report fewer depressive symptoms and better quality of life across diverse settings, including rural China, Latin-American community samples, and older adults with chronic diseases [29,50,53]. In this sense, our findings are consistent with the broader literature and highlight that perceptions of emotional connectedness and support remain central to psychological well-being, even in small rural communities, where extended family networks are often assumed to be strong.

### 4.2. Social Support as a Mediator: Socio-Emotional Pathways

The simple mediation analysis indicated a significant indirect effect of stress on depressive symptoms through global perceived social support: higher stress was associated with lower perceived support, which in turn predicted higher depression scores. Similar mediation patterns have been documented among family caregivers, older adults with chronic illness, and community-dwelling seniors, where social support or related psychosocial resources explain part of the stress–mental-health relationship [54,55]. In rural Chinese older adults, for example, depressive symptoms have been shown to mediate the effect of low social support on quality of life, underscoring the central role of mood in linking social resources to broader well-being [56].

Our parallel multiple mediation model adds nuance by showing that not all support dimensions contribute equally to the indirect pathway. Only positive social interaction and affectionate support produced significant, specific indirect effects, whereas emotional/informational and instrumental support did not. This pattern aligns with prior work indicating that companionship, enjoyable shared activities, and affective closeness play a particularly salient role in late-life mental health, beyond more cognitive or advisory forms of support [25,57].

One interpretation is that chronic stressors (e.g., health limitations, financial strain) may reduce opportunities for pleasant social activities and affectionate exchanges; the perceived loss or unavailability of these experiences may be what most strongly undermines psychological well-being. This interpretation is consistent with longitudinal evidence that social resources can deteriorate under chronic strain, thereby contributing to emotional difficulties. For example, studies in older adults have found that initial levels of social support predict subsequent depressive symptoms, in part because stress-related increases in daily hassles erode supportive exchanges over time. Such findings align with stress-process models in which social resources function as secondary stressors when they become depleted or less available under sustained adversity [58].

### 4.3. Social Support as a Buffer: The Specific Role of Instrumental Help

In contrast to the classic stress-buffering hypothesis, global perceived social support did not significantly moderate the association between stress and depressive symptoms in our sample. This pattern aligns with prior research in older adult populations, which shows robust main effects of social support on depressive outcomes, yet limited or inconsistent evidence of interaction effects [58]. At the same time, other work has identified a buffering impact in alternative domains of well-being, such as life satisfaction [49]. However, when examining specific dimensions of support, our analyses showed that tangible (instrumental) support significantly moderated the stress–depression link: among older adults who reported higher levels of practical assistance, the association between stress and depressive symptoms was weaker.

Instrumental support, help with activities of daily living, accessing services, or managing treatment, has been identified as particularly important for maintaining mental health among caregivers and chronically ill older adults [59,60]. Our findings extend this evidence by suggesting that, in rural older adults, practical assistance may be the dimension of support most likely to buffer the emotional impact of stress, even though socio-emotional forms of support are more prominent in mediating pathways. In resource-constrained settings, instrumental support may directly reduce exposure to stressors (e.g., transportation, financial strain, or health-care access), thereby limiting the extent to which perceived stress translates into depressive symptoms [61].

The absence of buffering for emotional, interactional, or affectionate support may reflect ceiling effects, if these dimensions are relatively high across the sample, or indicate that socio-emotional support primarily operates as a background resource influencing baseline levels of depression rather than dynamically altering the stress–depression slope. Mixed findings across the broader literature suggest that stress-buffering effects depend on the match between the type of support and the stressors faced, as well as on cultural norms surrounding the receipt of help [49,58,62,63,64].

Collectively, these results contribute to theory by integrating stress-process and stress-buffering models within a functional (dimension-specific) view of perceived support. Consistent with stress-process theory, higher perceived stress was linked to lower socioemotional support (emotional/informational, affectionate, and positive social interaction), which in turn related to greater depressive symptoms, suggesting that erosion of social resources may partially transmit stress to depressive symptomatology [28]. In parallel, the observed moderation of tangible support aligns with the buffering hypothesis, indicating that concrete assistance can attenuate the impact of stressors on depressive symptoms when daily demands are high [26].

This functional specificity is consistent with evidence that emotional support shows more consistent inverse associations with depressive symptoms in later life than instrumental support [24,25] and with mechanistic accounts emphasizing that distinct supportive functions operate through different psychological pathways (e.g., stress appraisal, coping, belonging, perceived control) [23]. Taken together, our findings suggest that relying solely on a global support score may mask meaningful heterogeneity and that specifying support dimensions can improve theoretical clarity when modeling psychosocial pathways to late-life depression.

### 4.4. Implications for Practice and Policy in Rural Low and Middle-Income Countries (LMIC) Settings

These findings have several implications for clinical practice and community-based programs serving rural older adults in LMIC. First, they suggest that interventions should simultaneously address socio-emotional and instrumental aspects of support. Programs that focus exclusively on emotional support, without reducing practical barriers in daily life, may be insufficient to lower stress-related depressive symptoms in contexts where poverty, multimorbidity, and geographic isolation are common.

Second, the mediating role of positive social interaction and affectionate support underscores the importance of community-based activities that foster enjoyable, supportive contact among older adults, such as social clubs, group exercise, cultural events, and faith-based gatherings. Such initiatives may strengthen social connection and reduce loneliness and perceived isolation—an especially relevant goal in the post-pandemic era.

Third, the specific buffering effect of instrumental support underscores the value of home-based services, caregiver training, and community health worker programs that provide concrete assistance with daily tasks, medication management, and access to health care. Using tools such as the MOS-SSS to profile social-support needs, primary-care teams can identify older adults who lack practical help and tailor interventions accordingly. Strengthening the links between primary health centers and local informal support networks may therefore be a promising strategy for improving mental health and overall well-being among rural older adults.

### 4.5. Limitations

Several limitations should be considered when interpreting these findings. An initial limitation is the cross-sectional design, which precludes causal inference and does not allow us to determine the temporal ordering of perceived stress, social support, and depressive symptoms. Bidirectional relationships are plausible; for example, depressive symptoms may erode perceived support and heighten stress perceptions. A further limitation concerns the use of convenience sampling from a single rural health center, which may limit the generalizability of the results to other rural communities or to urban older-adult populations within the same country.

An additional limitation is that, although we statistically adjusted for several sociodemographic and health-related covariates, residual confounding by unmeasured factors, such as personality traits, subclinical cognitive impairment, exposure to lifetime adversity or violence, and quality or continuity of healthcare, cannot be ruled out. Moreover, all variables were assessed through self-report questionnaires, which may be subject to recall bias and social desirability, particularly in domains related to mental health and family relationships. We also did not directly assess loneliness or objective social isolation/network characteristics, which limits our ability to disentangle perceived social connection from structural isolation.

Finally, although the sample size was adequate for the planned mediation and moderation analyses, some interaction effects, especially those involving individual support dimensions, may have been underpowered to detect small buffering effects. MOS-SSS dimensions were moderately collinear (VIF up to 6.38), which may have increased standard errors in models including multiple dimensions simultaneously; therefore, non-significant dimension-specific indirect effects should be interpreted cautiously. Larger, preferably longitudinal studies are needed to clarify when and for whom specific forms of social support primarily mediate versus buffer the association between stress and depressive symptoms in later life.

## 5. Conclusions

In this community sample of rural older adults from northern Peru, perceived stress was closely linked to depressive symptoms and to multiple facets of perceived social support. Older adults who reported higher stress tended to feel less supported and showed more depressive symptomatology, underscoring the central role of social connections in late-life psychological well-being in resource-constrained rural settings.

Our analyses indicate that social support protects mental health through two complementary pathways. Global perceived support, and especially its socio-emotional components, namely, positive social interaction and affectionate support, partly explained the association between stress and depressive symptoms, suggesting that companionship and warm, emotionally meaningful ties are key mechanisms through which stress translates into poorer well-being. At the same time, instrumental support emerged as the only dimension that buffered the impact of stress on depression, pointing to the importance of practical assistance with daily tasks, finances, and access to health care.

Taken together, these findings suggest that efforts to promote mental health in rural older adults in low- and middle-income countries should not focus exclusively on either emotional or practical support. Instead, interventions and policies are likely to be most effective when they simultaneously strengthen opportunities for enjoyable, supportive social interaction to enhance social connection and reduce loneliness and ensure that older adults have reliable access to concrete, instrumental help. Future longitudinal and intervention studies are needed to confirm these patterns over time and to identify which combinations of socio-emotional and instrumental support are most effective in reducing stress-related depression in later life.

## Figures and Tables

**Table 2:** Descriptive Statistics and Spearman Correlations Among Study Variables.

Variables	1	2	3	4	5	6	7	Median (IQR)
1. Stress	-							7.50 (3.00)
2. Depression	0.644 *	-						3.50 (4.50)
3. Social support	−0.511 *	−0.706 *	-					62.00 (23.25)
4. Emotional/informational support	−0.490 *	−0.713 *	0.951 *	-				27.00 (11.00)
5. Tangible (instrumental) support	0.090	0.066	0.357 *	0.170 *	-			8.00 (5.00)
6. Positive social interaction	−0.571 *	−0.749 *	0.909 *	0.866 *	0.116	-		14.50 (6.00)
7. Affectionate support	−0.571 *	−0.724 *	0.875 *	0.837 *	0.067	0.853 *	-	12.00 (6.00)

Note: * *p* < 0.05. IQR = interquartile range.

**Table 3:** Simple mediation of the association between perceived stress and depressive symptoms by global perceived social support.

Effect	Path/Outcome	B	SE	t	*p*	95% CI (LL, UL)	Standardized β
a	Stress → Social support (Social support as outcome)	−3.62	0.55	−6.55	<0.001	[−4.71, −2.53]	−0.47
b	Social support → Depression (Depression as outcome, controlling Stress + covariates)	−0.12	0.01	−9.27	<0.001	[−0.15, −0.09]	−0.53
c (total)	Stress → Depression (total effect)	1.03	0.11	9.30	<0.001	[0.81, 1.25]	0.60
c’ (direct)	Stress → Depression (direct effect, controlling Social support + covariates)	0.60	0.10	5.94	<0.001	[0.40, 0.80]	0.35
ab (indirect)	Stress → Social support → Depression (bootstrapped indirect effect)	0.43	0.08	—	—	[0.28, 0.60]	0.25

Note: Model fit: Social support model R^2^ = 0.23; Depression model R^2^ = 0.60; PROCESS Model 4 with 10,000 bootstrap samples; models adjusted for age, sex, marital status, educational level, economic level, and chronic medical condition (N = 166). CI = confidence interval.

**Table 4:** Moderation of the association between perceived stress and depressive symptoms by global perceived social support.

Predictor	B	SE	t	*p*	95% CI (LL, UL)
Stress	0.62	0.11	5.80	<0.001	[0.41, 0.83]
Global social support	−0.12	0.01	−9.23	<0.001	[−0.15, −0.09]
Stress × Social support	0.00	0.01	0.58	0.562	[−0.01, 0.02]

Note: Model fit: R = 0.78, R^2^ = 0.60, F(9, 156) = 26.29, *p* < 0.001. PROCESS Model 1 with 10,000 bootstrap samples; outcome is depressive symptoms (GDS-15 total score). Model adjusted for age, sex, marital status, educational level, economic level, and chronic medical condition (N = 166). CI = confidence interval.

**Table 5:** Parallel multiple mediation of the association between perceived stress and depressive symptoms by social support dimensions.

Mediator (Social Support Dimension)	Indirect Effect (B)	BootSE	95% BootCI (LL, UL)	Completely Standardized Indirect Effect	BootCI for Standardized Effect
Emotional/informational support	0.1420	0.0860	[−0.0197, 0.3198]	0.0819	[−0.0113, 0.1798]
Tangible (instrumental) support	0.0064	0.0160	[−0.0244, 0.0418]	0.0037	[−0.0141, 0.0240]
Positive social interaction	0.2929	0.1316	[0.0641, 0.5729]	0.1689	[0.0374, 0.3236]
Affectionate support	0.2245	0.1027	[0.0346, 0.4383]	0.1295	[0.0200, 0.2503]
Total indirect effect (all dimensions combined)	0.6658	0.1049	[0.4627, 0.8765]	0.3840	[0.2808, 0.4846]

Note: PROCESS Model 4 with 10,000 bootstrap samples; outcome is depressive symptoms (GDS-15 total score). Model adjusted for age, sex, marital status, educational level, economic level, and chronic medical condition (N = 166). Indirect effects represent the effect of perceived stress on depressive symptoms operating through each social support dimension or their combined total. BootCI = percentile bootstrap confidence interval based on 10,000 resamples.

**Table 6:** Moderation of the Stress–Depression Association by Social-Support Dimensions.

Moderator (Social Support Dimension)	B (Stress × Support)	SE	t	*p*	95% CI (LL, UL)
Emotional/informational support	0.0032	0.0124	0.26	0.797	[−0.0213, 0.0277]
Tangible (instrumental) support	−0.0827	0.0370	−2.24	0.027	[−0.1558, −0.0096]
Positive social interaction	−0.0005	0.0235	−0.02	0.984	[−0.0469, 0.0460]
Affectionate support	0.0082	0.0264	0.31	0.757	[−0.0440, 0.0604]

Note: PROCESS Model 1 with 10,000 bootstrap samples; outcome is depressive symptoms (GDS-15 total score). Each model includes perceived stress as the focal predictor (X), one social support dimension as the moderator (W), and age, sex, marital status, educational level, economic level, and chronic medical condition as covariates (N = 166). Coefficients represent the interaction between perceived stress and each social support dimension predicting depressive symptoms. Negative coefficients indicate that higher levels of the support dimension attenuate the association between stress and depressive symptoms. CI = confidence interval.

## Data Availability

The data that support the findings of this study are not publicly available due to ethical and privacy restrictions, as they contain potentially identifiable information about older adults living in a small rural community. De-identified data may be made available from the corresponding author upon reasonable request and subject to approval by the Research Ethics Committee.

## Supplementary Materials

The following supporting information can be downloaded at: https://www.mdpi.com/article/10.3390/healthcare14030336/s1, Table S1: MOS-SSS item-level diagnostics (corrected item–total correlation and Cronbach’s α if item deleted).

## Abbreviations

The following abbreviations are used in this manuscript:

GDS-15	Geriatric Depression Scale-15
IQR	Interquartile range
LMIC	low- and middle-income countries
MOS-SSS	Medical Outcomes Study Social Support Survey
PSS-4	Perceived Stress Scale-4
SPSS	Statistical Package for the Social Sciences
VIF	variance inflation factor
WHO	World Health Organization

## References

[1] Goodman-Palmer D., Ferriolli E., Gordon A.L., Greig C., Hirschhorn L.R., Ogunyemi A.O., Usmani B.A., Yohannes T., Davies J. (2023). Health and Wellbeing of Older People in LMICs: A Call for Research-Informed Decision Making. Lancet Glob. Health.

[2] World Health Organization Ageing and Health. https://www.who.int/news-room/fact-sheets/detail/ageing-and-health.

[3] GBD 2019 Mental Disorders Collaborators (2022). Global, Regional, and National Burden of 12 Mental Disorders in 204 Countries and Territories, 1990-2019: A Systematic Analysis for the Global Burden of Disease Study 2019. Lancet Psychiatry.

[4] Hu T., Zhao X., Wu M., Li Z., Luo L., Yang C., Yang F. (2022). Prevalence of Depression in Older Adults: A Systematic Review and Meta-Analysis. Psychiatry Res..

[5] World Health Organization Mental Health of Older Adults. https://www.who.int/news-room/fact-sheets/detail/mental-health-of-older-adults.

[6] Lin W., Zhang D., Wang Y., Zhang L., Yang J. (2024). Analysis of Depression Status and Influencing Factors in Middle-Aged and Elderly Patients with Chronic Diseases. Front. Psychol..

[7] Liu H., Zhou Z., Fan X., Shen C., Ma Y., Sun H., Xu Z. (2023). Association Between Multiple Chronic Conditions and Depressive Symptoms Among Older Adults in China: Evidence From the China Health and Retirement Longitudinal Study (CHARLS). Int. J. Public Health.

[8] Zhao S., Han L., Liu Y., Rui X. (2025). Investigation and Analysis of Mental Health Status of the Older Adult in Western Rural Areas. Front. Public Health.

[9] Lee J., Lee J., Hwang S., Chung M.-K., Park J.Y., Shin T., Lee K.-J., Lim H.-S., Urtnasan E., Kim M.-H. (2024). Longitudinal Examination of Stress and Depression in Older Adults Over a 2-Year Period: Moderation Effect of Varied Social Support Measures. Depress. Anxiety.

[10] Cristóbal-Narváez P., Haro J.M., Koyanagi A. (2022). Longitudinal Association between Perceived Stress and Depression among Community-Dwelling Older Adults: Findings from the Irish Longitudinal Study on Ageing. J. Affect. Disord..

[11] Sowan W., Rutin R., Cohen M. (2023). Chronic Stressors, Coping Strategies, and Depressive Symptoms: A Comparison across Older Age Groups. Stress Health.

[12] Pereira I.M., Almeida T.V., Ribeiro A.Q., de Mendonça E.T., de Oliveira D.M., Cotta R.M.M., Cavalcante R.B., Moreira T.R. (2024). Stress, Depression, and Anxiety Symptoms in Older Adults: Temporal Trend and Relationship with COVID-19. Salud Ment..

[13] Du Q., Yao M., Wang W., Wang J., Li S., Lu K., Li C., Wei Y., Zhang T., Yin F. (2025). Association Between Multimorbidity and Depression in Older Adults: Evidence From Six Large Longitudinal Cohorts. Am. J. Geriatr. Psychiatry.

[14] Mohd T.A.M.T., Yunus R.M., Hairi F., Hairi N.N., Choo W.Y. (2019). Social Support and Depression among Community Dwelling Older Adults in Asia: A Systematic Review. BMJ Open.

[15] Bélanger E., Ahmed T., Vafaei A., Curcio C.L., Phillips S.P., Zunzunegui M.V. (2016). Sources of Social Support Associated with Health and Quality of Life: A Cross-Sectional Study among Canadian and Latin American Older Adults. BMJ Open.

[16] Tang L., Wang D., Bai Z., Zhu Y., Chen R. (2022). Relationship between Social Support and Depression among Older People from Elderly Care Social Organizations in Anhui Province, China. Rev. d’Épidemiol. Santé Publique.

[17] Gurrapu R., Ammapattian T., Antony S. (2024). Perceived Social Support, Loneliness, and Depression among Elderly Living in Old-Age Homes. J. Fam. Med. Prim. Care.

[18] Carrasco M., Fernandez M., Alexander E., Herrara S. (2021). Loneliness in Older Chilean People: Importance of Family Dysfunction and Depression. Int. J. Ment. Health Promot..

[19] Steinhoff P., Reiner A. (2024). Physical Activity and Functional Social Support in Community-Dwelling Older Adults: A Scoping Review. BMC Public Health.

[20] Ricciardi E., Spano G., Tinella L., Lopez A., Clemente C., Bosco A., Caffò A.O. (2023). Perceived Social Support Mediates the Relationship between Use of Greenspace and Geriatric Depression: A Cross-Sectional Study in a Sample of South-Italian Older Adults. Int. J. Environ. Res. Public Health.

[21] Doreste-Mendez R., Oberlin L.E., Ilieva I., Chen S.Z., Gunning F.M., Solomonov N. (2023). Perception of Social Support and Cognitive Performance in Older Adults With Depression. JAMA Netw. Open.

[22] Arias-Monsalve A.M., Arias-Valencia S., Rubio-León D.C., Aguirre-Acevedo D.-C., Re Tifo Re Tifo L., Estrada Cortes J.A., Paredes Arturo Y.V. (2022). Factors Associated with Happiness in Rural Older Adults: An Exploratory Study. Int. J. Psychol. Res..

[23] Thoits P.A. (2011). Mechanisms Linking Social Ties and Support to Physical and Mental Health. J. Health Soc. Behav..

[24] Gariépy G., Honkaniemi H., Quesnel-Vallée A. (2016). Social Support and Protection from Depression: Systematic Review of Current Findings in Western Countries. Br. J. Psychiatry.

[25] Samtani S., Mahalingam G., Lam B.C.P., Lipnicki D.M., Numbers K., Lima-Costa M.F., Blay S.L., Costa E.C., Xiao S., Reidel-Heller S. (2025). Emotional and Instrumental Social Support and Older Adults’ Depressive Symptoms: Collaborative Individual Participant Data Meta-Analysis of 11 Population-Based Studies of Aging. Am. J. Epidemiol..

[26] Cohen S., Wills T.A. (1985). Stress, Social Support, and the Buffering Hypothesis. Psychol. Bull..

[27] Buchwald P. (2017). Social Support. Reference Module in Neuroscience and Biobehavioral Psychology.

[28] Pearlin L.I., Menaghan E.G., Lieberman M.A., Mullan J.T. (1981). The Stress Process. J. Health Soc. Behav..

[29] Li D., Jie J.-H., Li H., Xia X.-M., Zhang Y., Yang Y., Xiang J., Zhuang H.-L. (2025). The Relationship between Social Support and Depression among Older Adults with Hypertension in Urban Communities: Mediating Effects of Coping Styles. Front. Psychiatry.

[30] García-Pérez A., Pineda A.E.G.-A., Sandoval-Bonilla B.A., Cruz-Hervert L.P. (2022). Prevalence and Factors Associated with the Depressive Symptoms in Rural and Urban Mexican Older Adults: Evidence from the Mexican Health and Aging Study 2018. Salud Pública México.

[31] Carvalho B.O., Castro-Costa É., Lima-Costa M.F., Filho A.I.d.L. (2024). Association between depressive symptoms and social support in a nationally representative sample of older adults (ELSI-Brasil). Braz. J. Psychiatry.

[32] Murillo-Llorente M.T., Caballero Coloma N., Tomás-Aguirre F., Tejeda-Adell M., Ventura I., Perez-Bermejo M. (2023). Analysis of the Psychosocial Sphere of Older Adults in Extreme Poverty in the Peruvian Amazon. Healthcare.

[33] Loret de Mola C., Stanojevic S., Ruiz P., Gilman R.H., Smeeth L., Miranda J.J. (2012). The Effect of Rural-to-Urban Migration on Social Capital and Common Mental Disorders: PERU MIGRANT Study. Soc. Psychiatry Psychiatr. Epidemiol..

[34] Cohen S. (1988). Perceived Stress in a Probability Sample of the United States. The Social Psychology of Health; The Claremont Symposium on Applied Social Psychology.

[35] Cruz G.J.H.S., Saavedra J.R.R., Samamé M.L.T. (2022). Propiedades psicométricas de la escala de estrés percibido en adultos de 20 a 35 años de la ciudad de Chiclayo. UCV Hacer.

[36] Dominguez-Lara S., Merino-Soto C., Torres-Villalobos G. (2022). Análisis Estructural y de Fiabilidad de La Escala de Estrés Percibido (PSS) En Profesionales de Enfermería Del Perú. Enfermería Clínica.

[37] Remor E. (2006). Psychometric Properties of a European Spanish Version of the Perceived Stress Scale (PSS). Span. J. Psychol..

[38] Yesavage J.A., Brink T.L., Rose T.L., Lum O., Huang V., Adey M., Leirer V.O. (1982). Development and Validation of a Geriatric Depression Screening Scale: A Preliminary Report. J. Psychiatr. Res..

[39] Erazo M., Fors M., Mullo S., González P., Viada C. (2020). Internal Consistency of Yesavage Geriatric Depression Scale (GDS 15-Item Version) in Ecuadorian Older Adults. INQUIRY.

[40] Gallardo-Peralta L.P., Rodríguez-Blázquez C., Ayala-García A., Forjaz M.J. (2020). Multi-Ethnic Validation of 15-Item Geriatric Depression Scale in Chile. Psicol. Reflex. Crit..

[41] Merino-Soto C., Núñez Benítez M.Á., Domínguez-Guedea M.T., Toledano-Toledano F., Moral de la Rubia J., Astudillo-García C.I., Rivera-Rivera L., Leyva-López A., Angulo-Ramos M., Flores Laguna O.A. (2023). Medical Outcomes Study Social Support Survey (MOS-SSS) in Patients with Chronic Disease: A Psychometric Assessment. Front. Psychiatry.

[42] Ramos-Vera C., Calle D., Collacso Fiesta H., Lamilla L.L., Serpa-Barrientos A., Saintila J. (2023). Psychometric Properties of the Peruvian Version of the MOS Scale for Social Support in Cancer Patients and Convergent Network with Quality of Life. Patient Prefer. Adherence.

[43] Streiner D.L. (2003). Starting at the Beginning: An Introduction to Coefficient Alpha and Internal Consistency. J. Pers. Assess..

[44] Tavakol M., Dennick R. (2011). Making Sense of Cronbach’s Alpha. Int. J. Med. Educ..

[45] Hayes A.F., Rockwood N.J. (2017). Regression-Based Statistical Mediation and Moderation Analysis in Clinical Research: Observations, Recommendations, and Implementation. Behav. Res. Ther..

[46] The World Bank (2023). Rising Strong: Peru Poverty and Equity Assessment.

[47] Bazo-Alvarez J.C., Bazalar-Palacios J., Bazalar J., Flores E.C. (2022). Mental Health among the Sugarcane Industry Farmers and Non-Farmers in Peru: A Cross-Sectional Study on Occupational Health. BMJ Open.

[48] Del Brutto O.H., Mera R.M., Rumbea D.A., Arias E.E., Sedler M.J. (2024). Does Escalating Violence and Associated Fear of Crime Worsen Psychological Well-Being in Community Dwellers Living in a Rural Setting? Results From the Atahualpa Project Cohort. J. Prim. Care Community Health.

[49] Adams T.R., Rabin L.A., Da Silva V.G., Katz M.J., Fogel J., Lipton R.B. (2016). Social Support Buffers the Impact of Depressive Symptoms on Life Satisfaction in Old Age. Clin. Gerontol..

[50] Li Z., Qin S., Zhu Y., Zhou Q., Yi A., Mo C., Gao J., Chen J., Wang T., Feng Z. (2025). Social Support Mediates the Relationship between Depression and Subjective Well-Being in Elderly Patients with Chronic Diseases: Evidence from a Survey in Rural Western China. PLoS ONE.

[51] Singh S., Shri N., Dwivedi L.K. (2022). An Association between Multi-Morbidity and Depressive Symptoms among Indian Adults Based on Propensity Score Matching. Sci. Rep..

[52] Wickrama K.A.S., Klopack E.T., O’Neal C.W. (2021). How Midlife Chronic Stress Combines with Stressful Life Events to Influence Later Life Mental and Physical Health for Husbands and Wives in Enduring Marriages. J. Aging Health.

[53] Hao R., Jin H., Zuo J., Wu Y., Sun X., Hu J. (2023). The Multiple Mediating Effect of Family Health and Perceived Social Support on Depressive Symptoms in Older Adults: A Cross-Sectional National Survey in China. J. Affect. Disord..

[54] Lee H.J., Seo J.M., Ahn S.H. (2017). The Role of Social Support in the Relationship between Stress and Depression among Family Caregivers of Older Adults with Dementia. J. Korean Acad. Nurs..

[55] Amin S.M., Khedr M.A., Tawfik A.F., Gamal Noaman Malek M., El-Ashry A.M. (2025). The Mediating and Moderating Role of Social Support on the Relationship between Psychological Well-Being and Burdensomeness among Elderly with Chronic Illness: Community Nursing Perspective. BMC Nurs..

[56] Wang J., Xue J., Jiang Y., Zhu T., Chen S. (2020). Mediating Effects of Depressive Symptoms on Social Support and Quality of Life among Rural Older Chinese. Health Qual. Life Outcomes.

[57] Lüscher J., Pauly T., Gerstorf D., Stadler G., Ashe M.C., Madden K.M., Hoppmann C.A. (2022). Having a Good Time Together: The Role of Companionship in Older Couples’ Everyday Life. Gerontology.

[58] Russell D.W., Cutrona C.E. (1991). Social Support, Stress, and Depressive Symptoms among the Elderly: Test of a Process Model. Psychol. Aging.

[59] Kent E.E., Mollica M.A., Dionne-Odom J.N., Ferrer R.A., Jensen R.E., Ornstein K.A., Smith A.W. (2020). Effect of Instrumental Support on Distress among Family Caregivers: Findings from a Nationally Representative Study. Palliat. Support. Care.

[60] Sambasivam R., Liu J., Vaingankar J.A., Ong H.L., Tan M.-E., Fauziana R., Picco L., Chong S.A., Subramaniam M. (2019). The Hidden Patient: Chronic Physical Morbidity, Psychological Distress, and Quality of Life in Caregivers of Older Adults. Psychogeriatrics.

[61] Fu D., Wang F., Gao B., Bai Q., Liu G., Zhu J. (2024). The Influence of Different Sources of Anticipated Instrumental Support on Depressive Symptoms in Older Adults. Front. Public Health.

[62] Pluut H., Ilies R., Curşeu P.L., Liu Y. (2018). Social Support at Work and at Home: Dual-Buffering Effects in the Work-Family Conflict Process. Organ. Behav. Hum. Decis. Process..

[63] Mojaverian T., Kim H.S. (2013). Interpreting a Helping Hand: Cultural Variation in the Effectiveness of Solicited and Unsolicited Social Support. Pers. Soc. Psychol. Bull..

[64] Campos B., Yim I.S., Busse D. (2018). Culture as a Pathway to Maximizing the Stress-Buffering Role of Social Support. Hisp. J. Behav. Sci..

