# Supplementary material for: Perceived Social Support and Well-Being: Mediation and Buffering of the Stress–Depression Link in Rural Older Adults
Source: Healthcare (Basel). 2026 Jan 29;14(3):336. doi: 10.3390/healthcare14030336 (PMC12896921; doi:10.3390/healthcare14030336)
Supplement: Supplementary file 1 [file healthcare-14-00336-s001.zip › healthcare-4104292-supplementary.pdf]

Table S1. MOS-SSS item-level diagnostics (corrected item–total correlation and Cronbach's α if item deleted)

Item	Dimension	Corrected item-total correlation	α if item deleted
ei1	Emotional/Informational	0.787	0.942
ei2	Emotional/Informational	0.782	0.942
ei3	Emotional/Informational	0.792	0.942
ei4	Emotional/Informational	0.742	0.943
ei5	Emotional/Informational	0.743	0.943
ei6	Emotional/Informational	0.752	0.943
ei7	Emotional/Informational	0.783	0.942
ei8	Emotional/Informational	0.824	0.941
ti1	Tangible (Instrumental)	0.088	0.953
ti2	Tangible (Instrumental)	0.430	0.949
ti3	Tangible (Instrumental)	0.377	0.949
ti4	Tangible (Instrumental)	0.329	0.950
a1	Affectionate	0.830	0.941
a2	Affectionate	0.749	0.943
a3	Affectionate	0.821	0.942
psi1	Positive Social Interaction	0.802	0.942
psi2	Positive Social Interaction	0.751	0.943
psi3	Positive Social Interaction	0.835	0.942
psi4	Positive Social Interaction	0.795	0.942

Note: Item codes indicate subscale and item order: ei = emotional/informational; ti = tangible (instrumental); a = affectionate; psi = positive social interaction.
